# Two-Stage Training Framework Using Multicontrast MRI Radiomics for *IDH* Mutation Status Prediction in Glioma

**DOI:** 10.1148/ryai.230218

**Published:** 2024-05-22

**Authors:** Nghi C. D. Truong, Chandan Ganesh Bangalore Yogananda, Benjamin C. Wagner, James M. Holcomb, Divya Reddy, Niloufar Saadat, Kimmo J. Hatanpaa, Toral R. Patel, Baowei Fei, Matthew D. Lee, Rajan Jain, Richard J. Bruce, Marco C. Pinho, Ananth J. Madhuranthakam, Joseph A. Maldjian

**Affiliations:** From the Departments of Radiology (N.C.D.T., C.G.B.Y., B.C.W., J.M.H., D.R., N.S., B.F., M.C.P., A.J.M., J.A.M.), Pathology (K.J.H.), and Neurologic Surgery (T.R.P.), The University of Texas Southwestern Medical Center, 5323 Harry Hines Blvd, Dallas, TX 75390; Department of Bioengineering, The University of Texas at Dallas, Richardson, Tex (B.F.); Departments of Radiology (M.D.L., R.J.) and Neurosurgery (R.J.), New York University Grossman School of Medicine, New York, NY; and Department of Radiology, University of Wisconsin–Madison, Madison, Wis (R.J.B.).

**Keywords:** Glioma, Isocitrate Dehydrogenase Mutation, *IDH* Mutation, Radiomics, MRI

## Abstract

**Purpose:**

To develop a radiomics framework for preoperative MRI-based prediction of isocitrate dehydrogenase (*IDH*) mutation status, a crucial glioma prognostic indicator.

**Materials and Methods:**

Radiomics features (shape, first-order statistics, and texture) were extracted from the whole tumor or the combination of nonenhancing, necrosis, and edema regions. Segmentation masks were obtained via the federated tumor segmentation tool or the original data source. Boruta, a wrapper-based feature selection algorithm, identified relevant features. Addressing the imbalance between mutated and wild-type cases, multiple prediction models were trained on balanced data subsets using random forest or XGBoost and assembled to build the final classifier. The framework was evaluated using retrospective MRI scans from three public datasets (The Cancer Imaging Archive [TCIA, 227 patients], the University of California San Francisco Preoperative Diffuse Glioma MRI dataset [UCSF, 495 patients], and the Erasmus Glioma Database [EGD, 456 patients]) and internal datasets collected from the University of Texas Southwestern Medical Center (UTSW, 356 patients), New York University (NYU, 136 patients), and University of Wisconsin–Madison (UWM, 174 patients). TCIA and UTSW served as separate training sets, while the remaining data constituted the test set (1617 or 1488 testing cases, respectively).

**Results:**

The best performing models trained on the TCIA dataset achieved area under the receiver operating characteristic curve (AUC) values of 0.89 for UTSW, 0.86 for NYU, 0.93 for UWM, 0.94 for UCSF, and 0.88 for EGD test sets. The best performing models trained on the UTSW dataset achieved slightly higher AUCs: 0.92 for TCIA, 0.88 for NYU, 0.96 for UWM, 0.93 for UCSF, and 0.90 for EGD.

**Conclusion:**

This MRI radiomics-based framework shows promise for accurate preoperative prediction of *IDH* mutation status in patients with glioma.

**Keywords:** Glioma, Isocitrate Dehydrogenase Mutation, *IDH* Mutation, Radiomics, MRI

*Supplemental material is available for this article.*

Published under a CC BY 4.0 license.

See also commentary by Moassefi and Erickson in this issue.

## Graphical abstract

**Figure:**
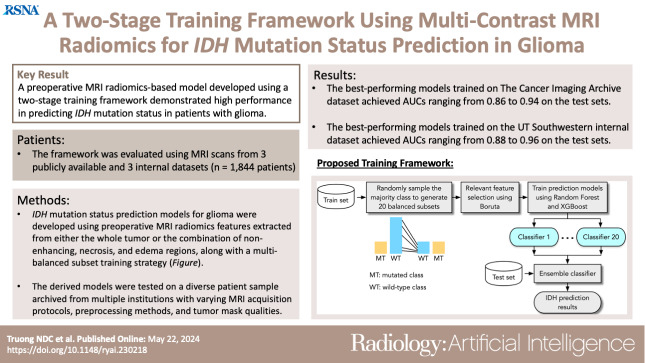

**Summary**

A preoperative MRI radiomics-based model developed using a two-stage training framework demonstrated high performance in predicting *IDH* mutation status in patients with glioma.

**Key Points**

■ *IDH* mutation status prediction models for glioma were developed using preoperative MRI radiomics features extracted from either the whole tumor or the combination of nonenhancing, necrosis, and edema regions, along with a multibalanced subset training strategy.
■ The best performing models trained on The Cancer Imaging Archive dataset achieved area under the receiver operating characteristic curve (AUC) values ranging from 0.86 to 0.94 when tested on internal and public datasets.
■ The best performing models trained on the University of Texas Southwestern Medical Center internal dataset achieved slightly higher AUC values, ranging from 0.88 to 0.96, on the internal and public test sets.

## Introduction

Glioma is a common and life-threatening type of brain tumor. The survival rates and responses to treatment of patients with glioma are influenced by the tumor’s genetic and histologic characteristics. Recent studies have identified isocitrate dehydrogenase (*IDH*) mutations as a crucial factor in the development and progression of glioma. Therefore, the World Health Organization updated the brain tumor classification in 2016 to include molecular marker diagnostics and classic histologic diagnostics (1). The World Health Organization also recommends determining the *IDH* status of patients with glioma to guide the selection of appropriate treatment therapies.

The detection of *IDH* mutation status is mainly based on genetic profiling of tumor tissue acquired through biopsy or surgical resection. However, depending on the accessibility of the mass, brain tumor resection may not be safe, and biopsy-based methods may cause complications. Therefore, noninvasive alternatives are important for obtaining genetic and histologic information. Radiomics is a novel technique to extract multidimensional features of a region of interest (ROI) from medical images (2). These features can be used to develop diagnostic or predictive models for outcomes of interest. Since MRI is currently included in routine clinical care for patients with glioma, radiomics features extracted from MRI have gained substantial interest as a promising method to predict *IDH* mutation status in these patients.

Radiomics features extracted from multicontrast MRI have been combined with machine learning techniques to develop models for predicting *IDH* mutation status (3–9). Some studies have focused on a specific histologic subtype of glioma, such as low-grade glioma (7,10,11) or high-grade glioma (8,12–14). Most radiomics-based models have been tested on relatively limited patient cohorts, either from The Cancer Imaging Archive (TCIA) dataset (3) or local data (12–14), primarily using the cross-validation method. A few radiomics-based studies extended assessment of the prediction model on an independent test set (7,8,15). Although these studies have reported *IDH* prediction accuracies ranging from 72% to 97%, an extensive evaluation of the prediction models on a larger patient sample is still needed to establish their effectiveness in clinical practice.

This study focused on developing *IDH* mutation prediction models in patients with glioma using preoperative MRI radiomics features and a two-stage training framework. Unlike previous studies that focused on specific tumor subcompartments, this study extracted radiomics features from either the whole tumor (WT) or the combination of nonenhancing tumor, necrosis, and edema regions (NET + NCR + ED). This inclusive approach allowed all patients to be included in the study, regardless of whether certain tumor subcompartments were absent (eg, enhancing tumor). Relevant radiomics features for *IDH* genotyping were identified through a feature selection algorithm and used in conjunction with machine learning techniques to build prediction models. Multiple models were trained using different balanced subsets resampled from the original imbalanced training dataset and then ensembled to build the final classifier. The derived models were tested on a diverse patient sample archived from multiple institutions with varying MRI acquisition protocols, preprocessing methods, and tumor mask qualities.

## Materials and Methods

### Datasets

This study used retrospective MRI scans from three publicly available and three internal datasets. The public datasets included data from The Cancer Genome Atlas (16) and the Ivy Glioblastoma Atlas (17), which were both downloaded from and together referred to as TCIA (18); the University of California San Francisco Preoperative Diffuse Glioma MRI dataset (UCSF) (19); and the Erasmus Glioma Database (EGD) (20). The internal datasets were collected from three geographically distinct institutions, namely the University of Texas Southwestern Medical Center (UTSW), New York University (NYU), and the University of Wisconsin–Madison (UWM). UTSW Institutional Review Board approval was obtained with a waiver of consent for the use of retrospective data or public datasets. All internal data were anonymized, and the study was compliant with the Health Insurance Portability and Accountability Act.

Data from the patients that met the following criteria were included in this study: (*a*) newly diagnosed with glioma; (*b*) *IDH* mutation status was available; (*c*) preoperative MRI scans with T1-weighted, postcontrast T1-weighted, T2-weighted, and T2-weighted fluid-attenuated inversion recovery sequences were available; and (*d*) tumor segmentation was available.

### Image Preprocessing and Multiregional Tumor Segmentation

The MRI data included in this study were collected from multiple sources and underwent distinct preprocessing tools due to the unavailability of raw data for uniform processing. However, the preprocessing pipeline of all datasets consisted of standardized steps commonly used for multimodal glioma analysis, including registering to a common anatomic space with a voxel resolution of 1 × 1 × 1 mm^3^, correcting for bias field distortion, coregistering MRI scans to a template atlas, and removing all nonbrain tissue (skull stripping) from the image. The federated tumor segmentation (FeTS) (21) tool was used to preprocess the TCIA and internal datasets. The UCSF dataset underwent preprocessing using multiple publicly available tools, including the Advanced Normalization Tools (22) and the brain masking tool (23). For the EGD dataset, all scans were registered using Elastix, version 5.0.0 (3CX) (24), and the skull was stripped by HD-BET (25).

Tumor segmentation masks for the TCIA and three internal datasets were obtained using the FeTS tool. FeTS segmented the tumor into three subcompartments: the necrotic tumor core (NCR, label 1), the NET and peritumoral edematous/invaded tissue (NET/ED, label 2), and the enhancing part of the tumor (ET, label 4). These automated masks were used directly to extract radiomics features without manual correction.

For the UCSF dataset, a different automated segmentation tool based on the multimodal brain tumor segmentation challenge algorithm was used to obtain automated tumor segmentation masks (26). These masks were then corrected manually by a group of annotators and approved by a neuroradiologist with more than 15 years of experience. However, the segmentation labels for the UCSF dataset were slightly different from those of the TCIA and internal datasets. The tumor in the UCSF dataset was segmented into three subcompartments: the NET and necrotic tumor core (NET/NCR, label 1), ED (ED, label 2), and enhancing tumor (ET, label 4).

Finally, for the EGD dataset, only WT masks were available. These masks were segmented either manually or automatically using a convolutional neural network–based method (27). The automated masks in the EGD dataset were not manually corrected.

### Radiomics Feature Extraction

The brain tumor radiomics features were extracted using the PyRadiomics Python package (28). To ensure that all patients were included in the study, regardless of the presence or absence of any tumor subcompartment, two ROIs were defined: the WT and the nonenhancing and edematous (NET + NCR + ED) region. Radiomics features were extracted from both ROIs for each of the four MRI sequences (T1-weighted, postcontrast T1-weighted, T2-weighted, and T2-weighted fluid-attenuated inversion recovery).

Different types of radiomics features were extracted from the brain tumor, including shape, first-order, and texture features. Specifically, we extracted 14 shape features, 18 first-order features, 22 gray-level co-occurrence matrix features, 16 gray-level run-length matrix features, 16 gray-level size zone matrix features, 14 gray-level dependence matrix features, and five neighboring gray-tone difference matrix features.

In addition to the original images, 12 derived images for each MRI sequence were also generated to extract additional radiomics features. These derived images were obtained using different filters, including wavelet filtering at four levels, Laplacian of Gaussian filtering with four levels of σ (from 2 to 5 mm), square, square root, logarithm, and exponential operators. Each of these derived images emphasizes different characteristics of the original images, thereby enriching the extracted radiomics features. In total, we extracted 1197 radiomic features from each ROI and each MRI sequence. The detailed description of the feature extraction process is presented in Appendix S1. PyRadiomics settings, as well as image types, feature classes, and feature names used for feature extraction, are summarized in Tables S3 and S4.

### Data Balancing

**Figure 1:**
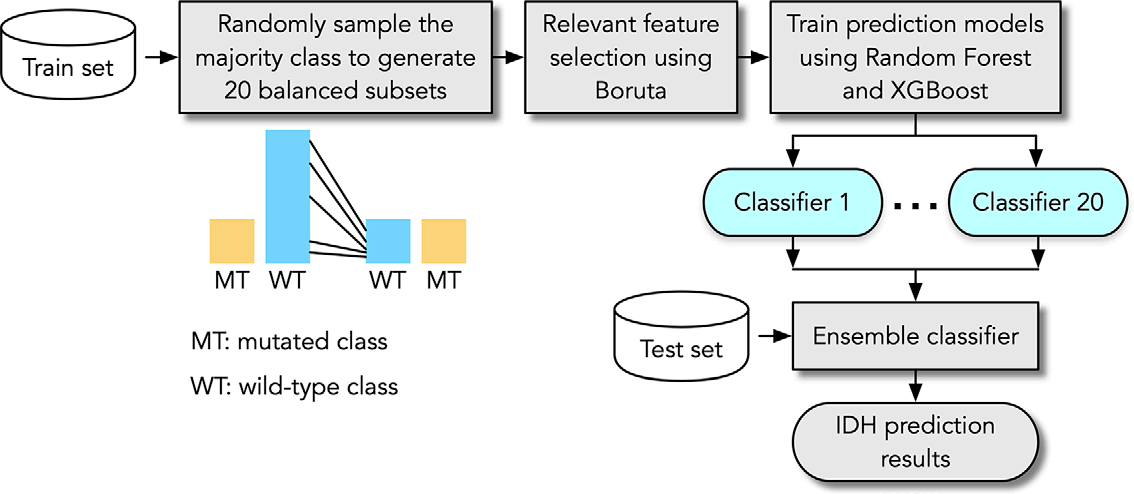
Flowchart of proposed MRI radiomics-based framework for predicting isocitrate dehydrogenase (*IDH*) mutation status in gliomas.

The overall prevalence of *IDH*-mutated tumors in our cohort was approximately 27%, which is lower than *IDH* wild type. Hence, to enhance classification performance on the unbalanced dataset, a two-stage training framework was implemented (Fig 1). In the first stage, multiple balanced training subsets were generated by randomly sampling the majority class (ie, *IDH* wild-type instances were randomly sampled to achieve a 1:1 ratio with the mutated cases). Then, a set of prediction models was trained using these subsets. In the second stage, we ensembled the prediction models by averaging the prediction probabilities of *IDH* mutation status. This approach was designed to produce an efficient classifier with improved accuracy and reduced bias toward the majority class.

### Feature Selection and *IDH* Mutation Classification

Boruta feature selection (29) was employed to identify the most relevant radiomics features for predicting *IDH* mutations. The Boruta method operates by generating random shadow features, which serve as a reference point for the actual features. The most relevant features were identified by assessing how frequently they outperform these shadow features. In our framework, Boruta was used on multiple balanced subsets of the training data. The most frequently selected features across these subsets were then considered the relevant feature set to train the classifier models. The radiomics features from each individual ROI (WT or [NET + NCR + ED]), as well as the combined features of both ROIs, were passed through Boruta to determine three sets of the most pertinent features.

The *IDH* mutation prediction models were then trained using two classifiers, random forest (30) and XGBoost (31). RF and XGBoost are both ensemble methods, which enable them to capture complex data relationships and handle high-dimensional feature spaces effectively. Additionally, they exhibit resilience to outliers, making them robust choices for this study. *IDH* prediction models were trained on multiple balanced subsets derived from either TCIA (*IDH* mutated, *n* = 94; *IDH* wild type, *n* = 133) or UTSW (*IDH* mutated, *n* = 102; *IDH* wild type, *n* = 256) data. These two datasets were selected as the training data since they had a sufficient number of mutated cases and were preprocessed by FeTS with the same tumor subregion annotations approach. The trained models were tested on all other held-out datasets. UTSW data were included in the test set for the models derived from the TCIA data used as the training set, and vice versa. The parameters for the Boruta feature selection method, as well as the random forest and XGBoost classifiers, can be found in Table S5.

### Statistical Analysis

The performance of the prediction models was assessed using several metrics, including accuracy, sensitivity, specificity, precision, F1 score, and the area under the receiver operating characteristic curve (AUC). To compute the AUC, the prediction probabilities of the mutated class were used to construct the receiver operating characteristic (ROC) curve. Various probability thresholds were applied to classify *IDH* status into either the mutated or wild-type class, resulting in a series of (1 – specificity, sensitivity) points forming the ROC curve. The AUC was calculated as the area under the ROC curve. The CI of the AUC was calculated using the DeLong method (32). The covariance of sensitivity and (1 – specificity) across all possible classification thresholds was first computed. This covariance was used to estimate the variance of the AUC. The CI was then derived using the standard normal distribution. Statistical metrics were calculated using the Scikit-learn package, version 1.2.1, in Python, version 3.8. No statistical significance testing was conducted in this study.

## Results

### Patient Characteristics

A total of 1844 patients across all datasets were included in this study (TCIA, 227 patients; UCSF, 495 patients; EGD, 456 patients; UTSW, 356 patients; NYU, 136 patients; and UWM, 174 patients). Table 1 summarizes the patient characteristics and *IDH* status of all datasets.

**Table 1:**
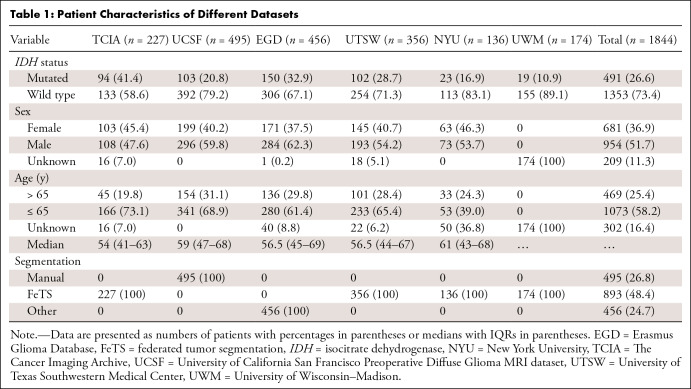
Patient Characteristics of Different Datasets

### Performance of Radiomics Models for *IDH* Mutation Status Prediction

The prediction performance of different models trained by TCIA and UTSW datasets is detailed in Tables 2 and 3, respectively. The 95% CIs for the AUC values are reported in Tables S7 and S8. Three models were compared: two models built from multicontrast features extracted from a single ROI (WT or [NET + NCR + ED]) and one model built from the combined features of these two ROIs. Because the EGD dataset had only WT masks, the reported results were only for the WT.

**Table 2:**
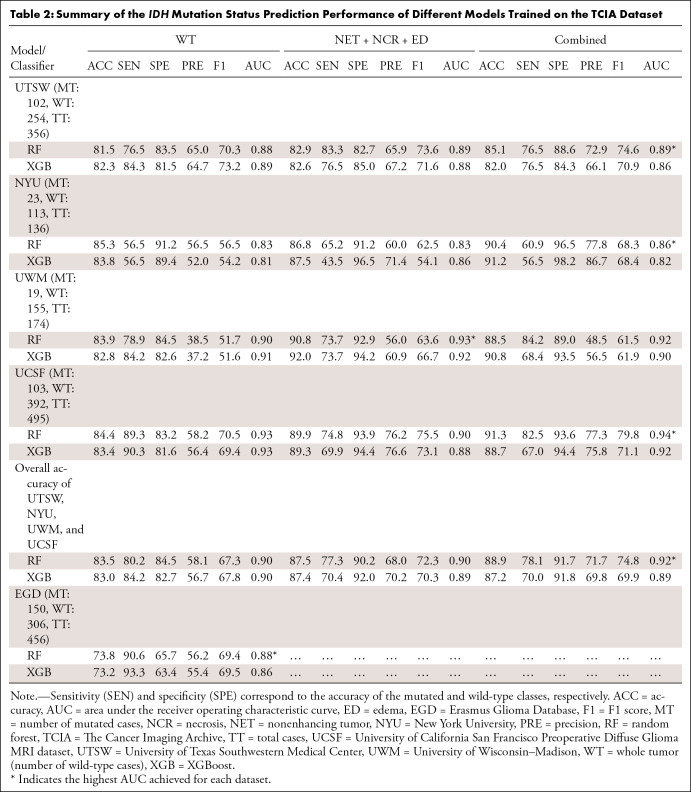
Summary of the *IDH* Mutation Status Prediction Performance of Different Models Trained on the TCIA Dataset

**Table 3:**
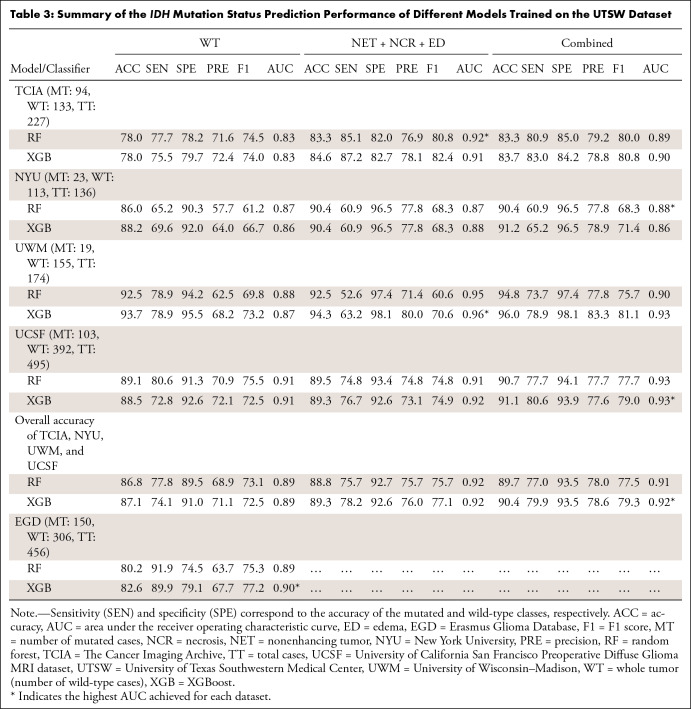
Summary of the *IDH* Mutation Status Prediction Performance of Different Models Trained on the UTSW Dataset

**Figure 2:**
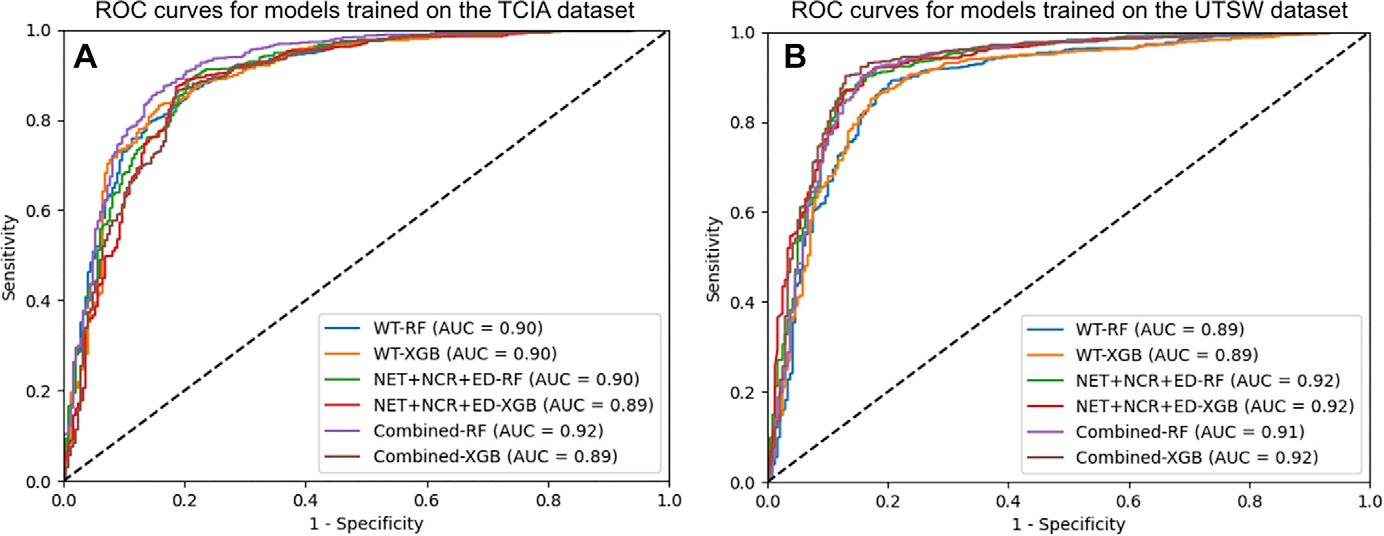
Receiver operating characteristic (ROC) curves for the random forest and XGBoost (XGB) models trained using relevant features from the whole tumor; the nonenhancing tumor (NET), necrosis (NCR), and edema (ED) region of interest; and the combined features from both regions of interest. **(A)** ROC curves for the combined test sets from the University of Texas Southwestern Medical Center (UTSW), New York University (NYU), University of Wisconsin–Madison, and University of California San Francisco Preoperative Diffuse Glioma MRI dataset (UCSF) obtained by models trained on The Cancer Imaging Archive (TCIA) dataset. **(B)** ROC curves for the combined test sets from TCIA, UTSW, NYU, and UCSF obtained by models trained on the UTSW dataset. AUC = area under the receiver operating characteristic curve, RF = random forest.

For models trained on the TCIA dataset, the highest AUC values were 0.89 (95% CI: 0.86, 0.92) for UTSW, 0.86 (95% CI: 0.80, 0.93) for NYU, 0.93 (95% CI: 0.89, 0.97) for UWM, 0.94 (95% CI: 0.92, 0.96) for UCSF, and 0.88 (95% CI: 0.85, 0.91) for EGD test sets, all obtained using the random forest classifier. The ROC curves for the combined test sets, including UTSW, NYU, UWM, and UCSF, are visually represented in Figure 2A. The models leveraging features extracted from both the WT and (NET + NCR + ED) ROIs appeared to exhibit slightly better performance.

Classifiers trained on the UTSW dataset appeared to perform slightly better than those trained with the TCIA dataset. Specifically, the highest AUC values were 0.92 (95% CI: 0.88, 0.95) for TCIA, 0.88 (95% CI: 0.81, 0.94) for NYU, 0.96 (95% CI: 0.93, 0.99) for UWM, 0.93 (95% CI: 0.91, 0.95) for UCSF, and 0.90 (95% CI: 0.87, 0.93) for EGD test sets, achieved mainly by XGBoost. Features extracted from the (NET + NCR + ED) ROI or the combination of features from both the WT and (NET + NCR + ED) ROIs generally led to improved AUC in most of the test datasets compared with using only the WT features. Model prediction performance on the EGD dataset showed a slight improvement when trained using the UTSW dataset compared with the TCIA dataset (AUC: 0.88 [95% CI: 0.85, 0.91] for TCIA and 0.90 [95% CI: 0.87, 0.93] for UTSW).

### Analysis of Radiomics Features Contributions

**Figure 3:**
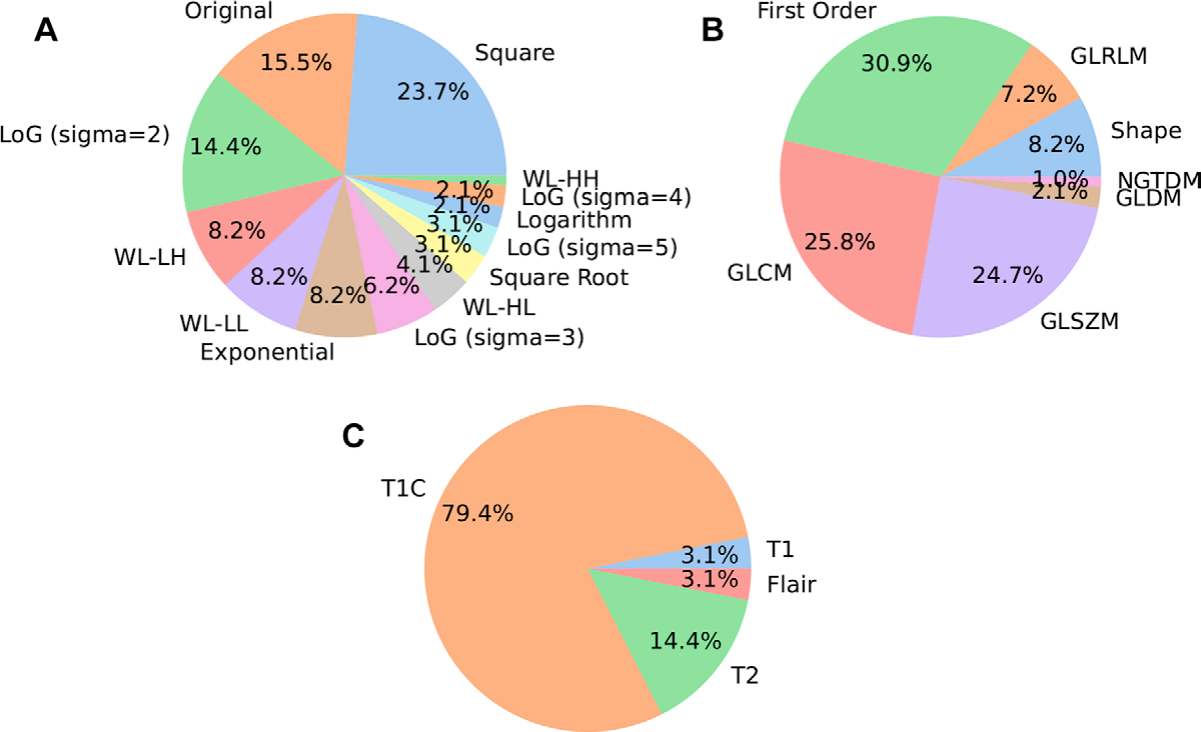
Summary of the most frequently selected image types, feature classes, and MRI sequences. **(A)** The most selected image types. **(B)** The most selected feature classes. **(C)** The percentage of MRI sequences identified as relevant features. GLCM = gray-level co-occurrence matrix, GLDM = gray-level dependence matrix, GLRLM = gray-level run-length matrix, GLSZM = gray-level size zone matrix, LoG = Laplacian of Gaussian, NGTDM = neighboring gray-tone difference matrix, WL-HH = wavelet filtering with high-pass filters, WL-HL = wavelet filtering with high-pass and low-pass filters, WL-LH = wavelet filtering with low-pass and high-pass filters, WL-LL = wavelet filtering with low-pass filters.

**Figure 4:**
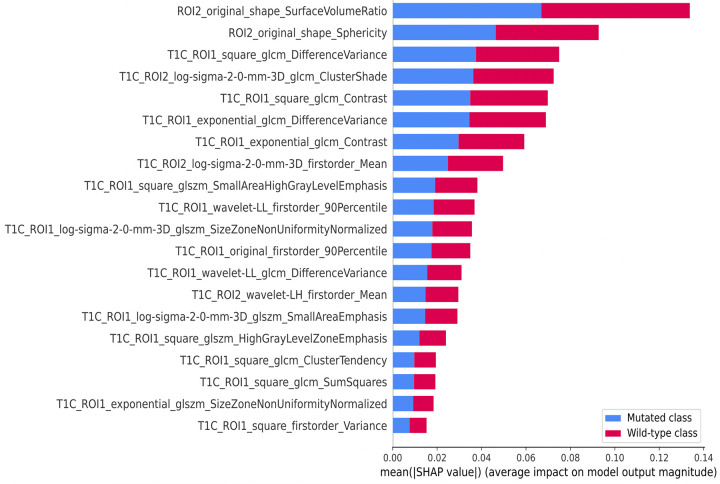
Chart of the top 20 relevant features with their importance scores measured by Shapley additive explanations (SHAP) values. The feature names are labeled as region of interest 1 (ROI1) and ROI2, representing the whole tumor and the nonenhancing tumor (NET), necrosis (NCR), and edema (ED) region of interests, respectively. glcm = gray-level co-occurrence matrix, glszm = gray-level size zone matrix, log = Laplacian of Gaussian, ROI = region of interest, wavelet-LH = wavelet filtering with low-pass and high-pass filters, wavelet-LL = wavelet filtering with low-pass filters.

Figure 3 provides an overview of the contributions of various image types, feature classes, and MRI sequences to the relevant feature set. Predominantly, the original images, squared images, and Laplacian of Gaussian images were the main contributors among the image types. Similarly, first-order statistics, gray-level co-occurrence matrix, and gray-level size zone matrix were the most selected feature classes. Furthermore, Figure 4 presents the most relevant features along with their important scores, which are quantified by the average of the absolute Shapley additive explanations (33) values, offering a clear insight into their impact.

## Discussion

We developed a preoperative MRI radiomics-based framework for predicting the *IDH* mutation status of gliomas. The prediction models were trained using the TCIA or UTSW datasets and tested on several independent test sets, including the NYU, UWM, UCSF, and EGD datasets. For the models trained on the TCIA dataset, the best overall AUC values were 0.92 (95% CI: 0.90, 0.93) on 1038 patients from the UTSW, NYU, UWM, and UCSF datasets, using combined features from both ROIs, and 0.88 (95% CI: 0.85, 0.91) on 456 patients of the EGD dataset, using the WT features. The models trained on the UTSW dataset appeared to perform slightly better than the TCIA-trained models likely due to the larger number of wild-type cases in the UTSW dataset and the two-stage training framework. The ROC curves showed improvement for the models trained by features from the (NET + NCR + ED) ROI or the combined features from both ROIs. Random forest and XGBoost algorithms performed comparably for both trained datasets.

Our model’s *IDH* prediction performance was assessed on an independent testing cohort comprising more than 1500 patients, marking it as one of the most extensive radiomics-based investigations. Previous studies have typically dealt with smaller datasets. For example, Lu et al (15) trained their radiomics-based prediction model on 306 patients from local institutions and tested it on 108 patients from The Cancer Genome Atlas, resulting in an AUC of 0.88. Li et al (3) employed a training cohort of 118 patients and a validation cohort of 107 patients, achieving the highest AUC of 0.96 when incorporating both radiomics and age as features. Some deep learning–based studies have incorporated slightly larger testing cohorts. van de Voort et al (34) trained their model on 1508 patients and tested on 221 patients from The Cancer Genome Atlas datasets, achieving an AUC of 0.90. Wu et al (35) attained an AUC of 0.87 when tested on 234 patients from an internal independent dataset. Our study, by leveraging a testing cohort exceeding 1500 patients, surpasses the prior studies in terms of dataset size, further bolstering the robustness and generalizability of our *IDH* prediction model.

Previous studies have revealed that training machine learning models on imbalanced data may result in prediction bias. Although various techniques have been used to address this issue, bias has remained a persistent problem. For instance, Li et al (13) addressed this problem by oversampling the mutated class, which comprised less than 10% of the cases in the training dataset, achieving an accuracy of 70% for the mutated class and 99% for the wild-type class. Another radiomics-based study (36) balanced the proportions of glioblastomas and anaplastic gliomas in the training dataset to account for the minority of *IDH*-mutated tumors rather than the proportions of mutated and wild-type tumors. This study reported an accuracy of 42% for the mutated class and 100% for the wild-type class.

In our study, we proposed a two-stage training framework that involves training multiple models on balanced subsets obtained by resampling the original imbalanced dataset. This approach resulted in 79.9% accuracy for the mutated class and 93.5% for the wild-type class, compared with the corresponding accuracy of 73.6% and 94.9% when training the models on balanced data using the synthetic minority oversampling technique (37), in which the mutated class was augmented to match the number of wild-type cases from the original training data. The full results obtained by the synthetic minority oversampling technique are presented in Tables S9 and S10. Thus, the two-stage training approach helped reduce bias and improve the accuracy of the mutated class.

The proposed radiomics-based framework extracted features from either the WT or a combination of NET, NCR, and ED subregions, making it suitable for any glioma grade. This approach differs from previous studies that focused on specific tumor subcompartments. By adopting this inclusive approach, all patients could be included in the study, regardless of whether certain tumor subcompartments were absent. Using these two tumor ROIs also enables the models to be trained and tested across various datasets with different definitions of the tumor subregions.

Figures 3 and 4 succinctly outline the distinctive contributions of different image types, feature classes, and MRI sequences to the relevant feature set. These features, derived from the combined region, offer a more holistic perspective of the tumor, potentially uncovering patterns that might be overlooked when focusing solely on individual tumor subcompartments. Notably, two shape features extracted from the (NET + NCR + ED) region exhibit high importance scores (Fig 4). The integration of these features has the potential to enhance the performance of the prediction models.

Our study had limitations. The main limitation of the radiomics-based approach is the need for a tumor mask, and the reliability of radiomic features depends on the accuracy of the tumor segmentation. However, brain tumor segmentation techniques have recently undergone substantial advancements, and many automatic brain tumor segmentation tools are now available. In our study, both the TCIA and internal data (UTSW, NYU, and UWM) were segmented using FeTS without manual correction. Although the radiomics features were extracted directly from the masks generated by FeTS, we achieved high prediction accuracies when testing on a large patient sample.

In conclusion, we present an MRI radiomics-based approach for predicting the *IDH* mutation status in both low-grade and high-grade gliomas. *IDH* prediction models were built based on a set of relevant radiomics features extracted from multicontrast MR images and two ROIs. The random forest and XGBoost methods were used as classifiers. A two-stage training strategy was adopted to address the unbalanced training data. The models were trained on either the TCIA or UTSW dataset and tested on the independent data, yielding promising prediction accuracy across a large and diverse patient sample. Future research may focus on improving performance of the *IDH* prediction models by incorporating patient demographic characteristics, as suggested in Jiang et al (38), and implementing a rigorous quality assurance procedure to ensure that the segmentation data meet rigorous standards of accuracy and reliability.
